# Expression of Intracellular Galectin-8 and -9 in Endometrial Cancer

**DOI:** 10.3390/ijms25136907

**Published:** 2024-06-24

**Authors:** Susanne Beyer, Maya Wehrmann, Sarah Meister, Fabian Trillsch, Franziska Ganster, Elisa Schmoeckel, Stefanie Corradini, Sven Mahner, Udo Jeschke, Mirjana Kessler, Alexander Burges, Thomas Kolben

**Affiliations:** 1 Department of Obstetrics and Gynecology, University Hospital, LMU Munich, Marchioninistr. 15, 81377 Munich, Germany; maya.wehrmann@med.uni-muenchen.de (M.W.); sarah.meister@med.uni-muenchen.de (S.M.); fabian.trillsch@med.uni-muenchen.de (F.T.); franziska.ganster@med.uni-muenchen.de (F.G.); sven.mahner@med.uni-muenchen.de (S.M.); udo.jeschke@uk-augsburg.de (U.J.); mirjana.kessler@med.uni-muenchen.de (M.K.); alexander.burges@med.uni-muenchen.de (A.B.); thomas.kolben@med.uni-muenchen.de (T.K.); 2 Institute of Pathology, TUM School of Medicine and Health, Trogerstraße 18, 81675 Munich, Germany; elisa.schmoeckel@tum.de; 3 Department of Radiation-Oncology, University Hospital, LMU Munich, 81377 Munich, Germany; stefanie.corradini@med.uni-muenchen.de; 4 Department of Obstetrics and Gynecology, University Hospital, Universitätsklinikum Augsburg, Stenglinstr. 2, 86156 Augsburg, Germany

**Keywords:** Gal-8, Gal-9, endometrial cancer, survival, prognosis, biomarker

## Abstract

Endometrial cancer (EC) is a common gynecological cancer worldwide. Treatment has been improved in recent years; however, in advanced stages, therapeutic options are still limited. The expression of galectins is increased in several tumor types and that they are involved in important cell processes. Large studies on endometrial cancer are still pending; Specimens of 225 patients with EC were immunohistochemically stained with antibodies for Gal-8 and Gal-9. Expression was correlated with histopathological variables. The cytosolic expression of both galectins is associated with grading and survival. Cytosolic Galectin-8 expression is a positive prognostic factor for overall survival (OS) and progression-free survival (PFS), while nuclear Gal-8 expression correlates only to OS. The cytosolic presence of Galectin-9 is correlated with a better prognosis regarding OS. Our results suggest that expression of both galectins is associated with OS and PFS in EC. Further studies are needed to understand the underlying molecular mechanisms.

## 1. Introduction

Endometrial cancer (EC) is the sixth most common cancer among women worldwide [1], and with an incidence of 382,069 cases and a mortality rate of 90,000 in 2018, it is very important for global health strategies [1]. The main risk for EC development is a high level of estrogen, related to, for example, early menarche, therapy with tamoxifen, nulliparity, diabetes, or obesity [2]. Because of the Western lifestyle, a further increasing incidence is expected [3]. Due to the identification of reliable molecular biomarkers, EC is no longer grouped in estrogen-dependent (Type I) and estrogen-independent (Type II) types [4], but is now classified according to the ProMisE-algorithm: this includes Mismatch repair (MMR-)-deficiency, Polymerase ε (POLE) mutated, p53 wildtype and p53 aberrant cancers [5,6]. Based on this knowledge, new therapeutic strategies regarding modalities are recommended [7]. In recurrent situations, checkpoint inhibitors like Pembolizumab were approved a few years ago [8]. However, there are still many patients, especially those with recurrent disease (about 15%) who have insufficient therapeutic options, so new markers are needed [9]. For this reason, oncological researchers focused on several cell and surface structures on EC, seeking new therapeutic targets, among them galectins.

Galectins (Gal) are proteins containing a highly conserved sequence and a β-galactoside-binding site, the carbohydrate recognition domain (CRD) [10]. Three subgroups can be determined depending on the molecular structure: Gal-1, Gal-2, Gal-7, Gal-10, Gal-13, Gal-14, Gal-15, and Gal-16 have one CRD and the tendency to dimerization (“prototype galectins”). “Tandem-repeat” galectins, including Gal-4, Gal-8, Gal-9, and Gal-12, have two CRDs which are connected by a linker peptide. Finally, Gal-3 is characterized by one C-terminal CRD and a nonlectin N-terminal domain (“chimera type”) [11,12,13].

Galectins can be found in the cytosol, in the nucleus, at membranes, and in the extracellular department [14,15]. They can interact with other non-galactosylated binding partners through the CRD or other parts of the galectin [16]. They bind glycans at the cell surface or extracellular matrix, but also non-carbohydrate ligands in the cytosol and nucleus. Galectins have a wide spectrum of effects in cell biology by their multiple binding capacities: they are involved in cell-cell- and cell-matrix-interaction as well as in the modulation of intracellular signaling and cellular functions [15,17].

**Figure 1:**
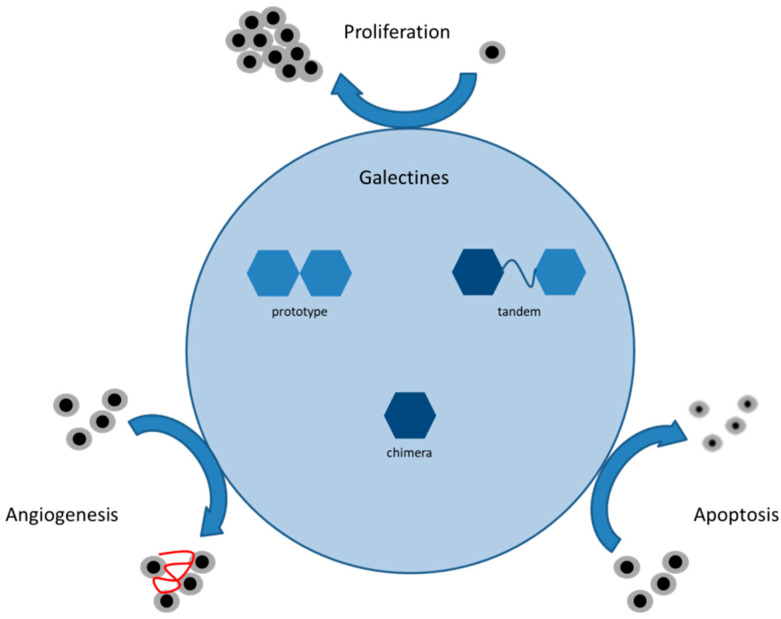
Summary of the function of different galectines in cancer.

An increased expression of galectins is described in several tumor types, suggesting a functional role of galectins in tumor growth and progression by affecting angiogenesis, invasion, metastasis, apoptosis, and also immune escape (see Figure 1). Depending on the type of cancer, they can have tumor-suppressive or tumor-activating effects [18]. Therefore, galectin expression is frequently usable as a prognostic marker for cancer patients‘ survival [19,20,21].

In gynecological cancers, two galectines are in the focus of research: Galectin-8 and -9, two tandem-repeat galectins. Both have been described to be involved in tumor progression:

Galectin-8 is expressed in normal cells throughout the body and modulates the interaction between leucocytes, platelets, and vascular endothelial cells. Therefore, it is involved in several physiological processes like hemostasis, infection, inflammation, and atherosclerosis [22]. One path of interaction is the activation of pro-inflammatory cytokines and chemokines like CCL2 and IL-6 [22]. In tumor cells, it is known to orchestrate cell adhesion and angiogenesis [22]. It seems to be a prognostic factor for multiple myeloma, ovarian, gastric, and urothelial cancer by immunohistochemistry, PCR, and Western blot [23,24,25,26]. In cervical cancer, Gal-8 expression was associated with negative N-status, lower FIGO status, and better relapse-free survival [27]. Similar results regarding survival were shown in ovarian cancer [23]. However, there is a wide variability of Gal-8 expression levels in malignant cells, depending on organ type, histological type, and tumor stage [22].

Galectin-9 plays multiple roles in tumor development. It is widely distributed in various cell types and regulates cell adhesion, for example of neutrophils and T-cells [22]. It is a prognostic factor in several entities of cancer. In many solid tumors, higher galectin-9 expression has been associated with lower tumor progression and better overall and progression-free survival [23,27,28,29]. An immunohistochemical examination of Gal-9 in cervical cancer showed a correlation to negative N-status, lower grading, and better overall survival [27]. Patients with ovarian cancer and a high Gal-9 expression also showed better survival data [23].

For both Gal-8 and Gal-9, the exact function in cancers is not completely understood.

A few studies have investigated the role of galectins in endometrial cancer. High expression of galectin-3 was correlated with cell adhesion, cell growth cycle, and cell proliferation in EC [30], but data are contradictory [31]: an immunohistochemical evaluation of gal-3 revealed a decreased galectin-3 expression as a sign of poor prognosis in EC [32]. Fewer studies exist regarding Galectin-7: it seems to increase cell migration in EC [33].

To our knowledge, no study has examined the role of Galectin-8, and only one study analyzed the role of Galectin-9 in 51 cases [30,34]. Given the fact that EC is seen as an immunogenic cancer, that Galectins seem to be involved in the mechanisms of immune escape, and that Gal-8 and Gal-9 have a prognostic value in other gynecological cancers, their prognostic value in EC still needs to be elucidated. Therefore, we examined the expression of Gal-8 and Gal-9 in EC in this study.

## 2. Results

### 2.1. Gal-8 Staining in Endometrial Cancer

**Figure 2:**
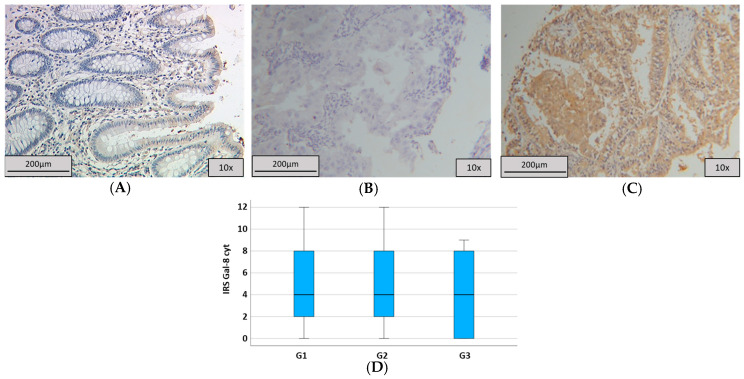
Galectin-8 staining. (**A**) Positive control of Galectin-8 staining in colon tissue with strong cytoplasmic expression in epithelial cells. (**B**) Low cytosolic Gal-8 expression with IRS = 2. (**C**) High cytosolic Gal-8 expression with IRS = 12. (**D**) Boxplot regarding grading and Gal-8 expression with a median of 4 in all subgroups.

As a positive control, we used non-pathological colon tissue that showed strong cytoplasmatic and nuclear expression in all epithelial cells (Figure 2A). We evaluated the staining of the cytoplasm and nucleus. In 77.8% of the samples, we found a cytoplasmic staining with Gal-8, with a median Immune Reactive Score (IRS) of 4 (17.3%), while 9.3% of the spots did not express Gal-8 (IRS = 0) in the cytoplasm. In total, 6.6% of the evaluated spots had an enhanced cytoplasmatic expression (IRS > 9). Regarding nuclear expression, the median IRS was 2 (13.8%) and a total of 54.6% of the samples showed Gal-8 nuclear staining. A total of 43.5% of all spots presented enhanced nuclear expression (IRS > 2), and 32.4% were not nuclearly stained (IRS = 0). A total of 118 samples (52.4%) showed cytosolic and nuclear staining simultaneously. In total, 12.9% of the samples could not be evaluated due to missing tumorous tissue. Examples of staining are shown in Figure 2B,C. We further investigated the correlation between Gal-8 and different clinical and histopathological markers like age, T-status, N-status, grading, and FIGO-status (Fédération Internationale de Gynécologie et d‘Obstétrique). For the distribution of these parameters, see Table 1. We detected a statistically significant association between lower grading and higher cytosolic Gal-8 expression when comparing G1 vs G2–3 (*p* = 0.029; *ρ* = −0.156 with *p* = 0.029, Figure 2D, Table 2), but not for G1 vs G2 vs G3. Although patients with all gradings had a median IRS of 4, the median was represented by 17.3% in G1, while it was only shown by 15.0% of the patients with G3.

Altogether, cytosolic Gal-8 was associated with grading (*p* = 0.029; Table 2). Regarding age (cytosolic: *p* = 0.791, nuclear: *p* = 0.458), N-status (*p* = 0.266 and *p* = 0.251), T-status (*p* = 0.217 and *p* = 0.509), or FIGO (*p* = 0.146 and *p* = 0.204), no significant difference associated with disease stage could be found (Table 2). No significant correlation between nuclear Gal-8 expression and histopathological markers was detected.

### 2.2. Gal-9 Staining in Endometrial Cancer

**Figure 3:**
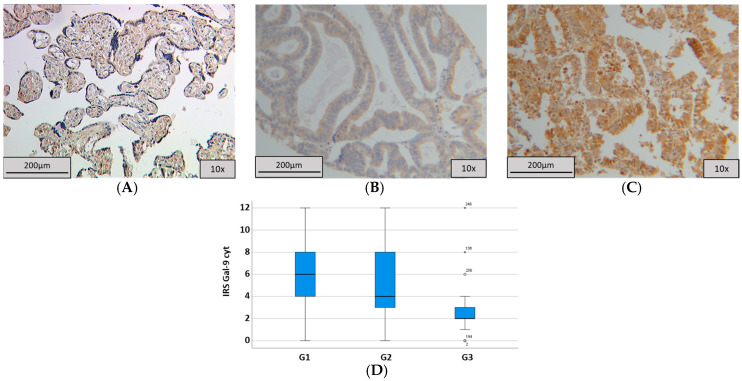
Galectin-9 staining. (**A**) Positive control of Galectin-9 staining in placenta tissue with strong cytoplasmic expression in trophoblastic cells. (**B**) Low cytosolic Gal-9 expression with IRS = 6. (**C**) High cytosolic Gal-9 expression with IRS = 12. (**D**) The summary regarding grading is shown in a boxplot. **^.^** = 1.5–3.0 Interquartile range; * = >3.0x Interquartile range.

To control the quality of the Gal-9 staining, we used normal placenta tissue, which is cytoplasmatic, but no nuclear expression could be found in >90% of the trophoblastic cells (Figure 3A).

The endometrial cancer samples showed cytoplasmic and weak nuclear expression of Gal-9. A total of 87.4% of the specimens were stained in the cytoplasm, while 4.0% had an IRS of 0. In total, 19.6% of the evaluated samples presented a median IRS of 4. Enhanced expression (IRS ≥ 3) was detected in 71.1% of the cases. In contrast, only 7.6% of samples were stained nuclear, while the median IRS of nuclear staining was 0 represented in 80.0% of the cells. However, 12.4% of the samples could not be evaluated due to missing tumor tissue. Simultaneous cytosolic and nuclear staining was detected in 25 cases (11.1%). Examples of staining are shown in Figure 3B,C. The investigation of the grading showed a statistically significant correlation between lower grading and altered cytosolic Gal-9 expression (*p* = 0.003; *ρ* = −0.215 with *p* < 0.001; Table 3). Patients with grading G1 presented a median IRS of 6, with 19.7% of samples showing this IRS. In contrast, samples of patients with G2 had a median IRS of 4 (26.0%) and samples with G3 showed a median IRS of 2 (35.0%; Figure 3D).

No statistically significant association could be detected for Gal-9 expression and age (Pearson coefficient for cytosolic Gal-9 = −0.006 with *p* = 0.935, nuclear = 0.051 with *p* = 0.466), pN-status (*p* = 0.227 and *p* = 0.057), pT-status (*p* = 0.516 and *p* = 0.509) or FIGO (*p* = 0.367 and *p* = 0.602; Table 3). No significant correlation between nuclear Gal-9 expression and histopathological markers was detected.

### 2.3. Correlation of Gal-8 and Gal-9 Expression

Spearman analysis of Gal-8 and -9 expression detected a correlation between cytoplasmic expression of the examined galectins. The nuclear and cytosolic expression of the same galectin also correlated, but not the nuclear Gal-8 expression with cytosolic Gal-9 expression or the other way around (Table 4).

### 2.4. Survival

The correlation of Gal-8 and -9 expression to overall survival (OS) and progression-free survival (PFS) of the patients was investigated. Tumors were distinguished in “high” and “low” expressing tumors using Receiver Operating Characteristic (ROC)-curve analysis.

#### 2.4.1. High Cytoplasmic Gal-8 Expression Is an Independent Positive Prognostic Marker for Overall Survival in Endometrial Cancer Patients

**Figure 4:**
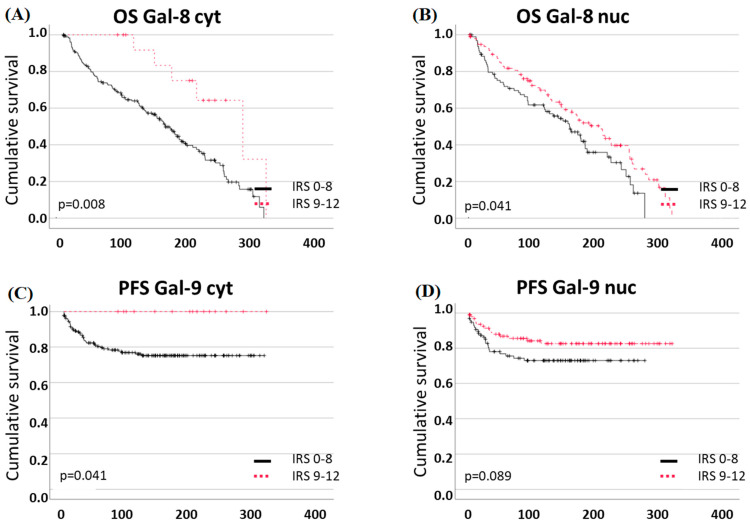
Gal-8 expression and survival: (**A**,**B**) High cytosolic and nuclear expression correlates to better OS rates. (**C**,**D**) High cytosolic expression correlates significantly with better PFS, but nuclear expression does not.

The analysis of OS showed that the expression of both cytosolic and nuclear Gal-8 is associated with better OS: High cytosolic expression of Gal-8 (IRS ≥ 9) was correlated with better prognosis in overall survival (*p* = 0.008, median OS cytosolic Gal-8 low: 160 months, cytosolic Gal-8 high: 280 months). For the Kaplan-Meier curve, see Figure 4A. Furthermore, patients with nuclear Gal-8 expression (IRS ≥ 2) in comparison to very few or no expression showed a better outcome (*p* = 0.041, median OS nuclear Gal-8 low: 157 months, nuclear Gal-8 high: 202 months; Figure 4B).

Multivariate COX regression analysis identified cytosolic Gal-8 expression as an independent prognostic factor for OS (*p* = 0.017). The age at diagnosis (*p* < 0.001) and grading (*p* = 0.042) were also independent prognosticators (Table 5).

We also investigated the correlation of Gal-8 for PFS: a significant difference in the outcome of patients with tumors of very high cytosolic Gal-8 expression (IRS ≥ 9) in comparison to lower Gal-8 expression was detected (*p* = 0.041, Figure 4C). This result matches the correlation of the galectins and the histopathological markers, as elevated Gal-8 expression went along with lower grading and better PFS rates. The COX regression did not confirm cytosolic Gal-8 expression as an independent prognostic factor regarding PFS (Table 6). Nuclear Gal-8 expression did not correlate significantly to PFS (*p* = 0.089; Figure 4D). However, subgroup analysis showed that Gal-8 expression in the nucleus had a prognostic relevance for improved PFS in the pT1 subgroup (but not in pT2/3/4, *p* = 0.039, Supplement Figure S1). No effects could be found in the subgroups of pN0/N1 or FIGO.

#### 2.4.2. High Cytoplasmic Gal-9 Expression Is a Positive Prognostic Marker for Overall Survival in Endometrial Cancer Patients

**Figure 5:**
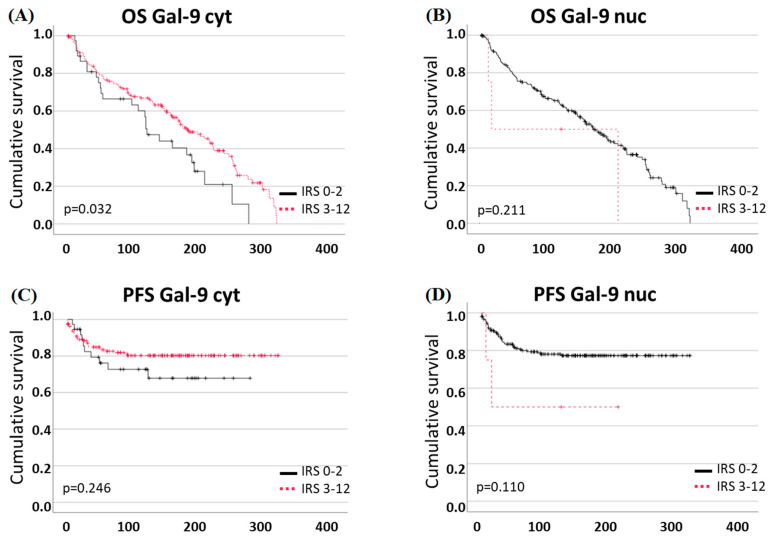
Gal-9 expression and survival: (**A**) High cytosolic expression correlates to better OS rates. (**B**) High nuclear expression is not correlated to OS. (**C**,**D**) Neither cytosolic nor nuclear Gal-9 correlation correlates to PFS.

Higher Gal-9 expression in the cytosol (IRS ≥ 3) was associated with a positive correlation to OS (*p* = 0.032, Figure 5A, median OS Gal-9 low: 120, Gal-9 high: 181 months). For nuclear Gal-9 expression and OS, as well as Gal-9 expression and PFS, no statistically significant correlation could be found (Figure 5B–D).

COX regression analysis concerning Gal-9 and OS showed that only age (*p* < 0.001), was an independent marker, but not the cytosolic or nuclear galectin-9 expression, nor other tested markers (Table 7). For PFS, none of the tested parameters in the COX regression analysis showed independence (Table 8).

Subgroup analysis showed that high cytosolic Gal-9 expression is a marker for improved PFS in the subgroup of lymph-node-negative patients (pN0, *p* = 0.035; Supplement Figure S2), but not in the subgroup of lymph-node positive-patients (pN1) or other subgroups concerning T-stage, grading or FIGO-stage.

## 3. Discussion

In this study, we examined the intracellular expression of Galectin-8 and -9 by immunohistochemistry in 225 endometrial cancer samples.

We detected an association of cytosolic Galectin-8 expression with low grading and better OS and PFS rates. Cytosolic expression of Gal-8 also correlated with high nuclear Gal-8 expression, which was associated with better OS. Galectin-9 expression correlated in its cytosolic presence with low grading and better OS rates. We also detected a correlation to nuclear Gal-9 expression as well as to cytosolic Gal-8 expression.

The family of galectins has an important impact on tumor biology by their effects on the cell cycle: they are involved in mechanisms like cell proliferation, apoptosis, metastasis, angiogenesis, and even immune response (Figure 1). Usually, they are located in the nucleus, but in cancer cells, Galectin-8 and -9 can be shifted to the cytoplasm, which we can confirm by a detected expression in both, the nucleus and cytoplasm [35,36].

Galectin-8 seems to be a modulator of tumor development and progression, being able to suppress or promote tumor growth. Its pro-angiogenetic and pro-migratory effects are mediated by the binding to the transmembranous glycoprotein CD166 in endothelial cells [37]. In Gal-8-expressing colorectal cancer cells, suppressive immune cells expanded, suggesting that Gal-8 regulates the tumor immune microenvironment [38]. Gal-8 also serves as a ligand for the immunosuppressive receptor LILRB4 [39]. Labrie et al. described that increased Gal-8/9 expression was correlated to poor response treatment in high-grade ovarian cancer patients [40].

On the other hand, Gal-8 is also known for its tumor-suppressive effects by inducing apoptosis and attenuating cell adhesions, for example in non-small cell lung carcinoma [41]. This is well examined in colon cancer cells, where Gal-8 expression leads to reduced migration and growth rate [36]. In contrast to Labrie et al., Schulz et al. revealed Gal-8 being a predictor for better OS and PFS in ovarian cancer by examining 156 cancer samples immunohistochemically [23]. Similar results were observed in squamous cell cervical cancer, where Gal-8 expression is also associated with better survival rates [27].

Our results suggest a correlation of high Galectin-8 expression with low grading and better survival rates, which match the already described pro-apoptotic effects of Galectin-8 and its effects in other cancers. Our observed data can be a base for further examinations. So far it seems clear, that the role of Gal-8 depends on the cancer tissue and its environment, whereas the details are still not understood. Further studies are needed, also considering the different isoforms of Gal-8 and their different effects depending on the tissue [42,43].

Galectin-9 was initially functionally described in Hodgkin-Lymphoma in 1997 [44]. It has several functions in tumor biology: besides an anti-metastatic effect, a pro-apoptotic potential is also described. Its pro-apoptotic effect is well examined in lymphoma cell lines [45] and ovarian cancer. Described mechanisms are caspase-dependent or mitochondria-mediated pathways [45,46,47]. Due to the significance of apoptosis regulation for cell fate and control of growth, its defects are often correlated to progression in cancer cells [48]. Additionally, Galectin-9 is known to be a modulator in inflammatory and immunological processes [29,49,50]. Several galectin-9 isoforms are known, resulting from alternative splicing and proteolytic processing of the Gal-9 gene (LGALS9), whereas some of these isoforms are involved in angiogenesis, as their expression is increased in activated and tumor endothelial cells [51].

Previous studies on endometrial cancer suggest Gal-9 expression to be associated with better survival rates and lower tumor stages in EC, as Sun et al. described in a small cohort [34]. Also, in cervical cancer and ovarian cancer, Gal-9 expression was correlated with better overall survival rates [23,27]. Its pro-apoptotic functions and its effects on cell adhesion can explain the positive outcome of tumors with higher Galectin-9 expression. Our results go along with these observations, as high Galectin-9 expression was correlated with better survival rates. An explanation might be Gal-9-induced apoptosis as observed in ovarian cancer cells by Jafari et al., but this has not been proven yet [46]. The human recombinant Gal-9, hG9NC (null), demonstrated anti-cancer activities, and induced apoptosis in hematological and gastrointestinal malignancies, making Gal-9 an interesting target for anti-cancer therapy [52].

The limit of this study is that it is not possible to distinguish the different isoforms of Galectin-8/-9 and our results are observations; the underlying molecular mechanisms have neither been examined nor understood.

Based on our results, further studies are needed to clarify its specific influence on the tumor biology of EC and to precisely determine the role of its different isotypes, which is technically not possible by immunohistochemistry so far. It would be also helpful to correlate Gal-8 and-9 expression to the new molecular classification according to The Cancer Genome Atlas (TCGA) [5,6]. Nevertheless, this is the first study, which examined the correlation of Galectin-8 and -9 with histopathological markers and survival data in a representative sample of endometrial cancer. Therefore, it may serve as a base for upcoming investigations of Gal-8 and -9 in EC.

## 4. Materials and Methods

### 4.1. Patients and Specimens

In total, 225 paraffin-embedded endometrial cancer samples were acquired from patients who underwent surgery from 1990 to 2002 in the Department of Obstetrics and Gynecology of the Ludwig-Maximilians-University of Munich. Inclusion criteria were (i) diagnosis of endometrioid endometrial cancer, (ii) surgery due to endometrioid endometrial cancer, and (iii) age > 18 years old. Patients < 18 years old and with non-endometrioid endometrial cancer were excluded due to the low number. The median age of the patients in the collective was 65.5 years, with a median overall survival (OS) of 131 months and a median progression-free survival (PFS) of 123 months. The distribution of histopathological markers is listed in Table 1. For positive controls of the immunohistochemical staining, we utilized colon and placenta tissue, both received from the Department of Obstetrics and Gynecology of the Ludwig-Maximilians-University of Munich. Clinical and follow-up data were provided by the Munich cancer registry (request from 18 January 2020) and retrieved from medical records. The histological subtype, staging (TNM-Classification 2010), and tumor grading were validated by a gynecological pathologist. OS was defined as the period of time from the date of first diagnosis until the date of death or last follow-up, PFS meant the period of time until local recurrence or metastasis was diagnosed.

### 4.2. Ethics Approval

All endometrial cancer specimens were collected for histopathological diagnostics. Clinical tests were completed when they were recruited for this study. Patient data were fully anonymized, and the authors were blinded for any information during experimental analyses. The study was conducted conforming to the Declaration of Helsinki and was approved by the local ethics committee of the Ludwig-Maximilians-University of Munich (reference number 19-249, 2019).

### 4.3. Immunohistochemistry

Paraffin-embedded and formalin-fixed samples were processed to tissue microarrays (TMAs) in the pathological institute of the Ludwig-Maximilians-University of Munich. The slides were deparaffinized in Roticlear (Carl Roth GmbH + Co. KG, Karlsruhe, Germany), a xylol-replacement medium. After washing the tissue in 100% ethanol, the endogenous peroxidase was blocked with 3% methanol/hydrogen peroxide (VWR International GmbH, Ismaning, Germany). The specimens were rehydrated in a descending series of alcohol (100%, 75%, 50%). Heat-induced antigen retrieval was performed by cooking the sections in a trisodium citrate buffer (pH = 6) in a pressure cooker at 100 °C for 5 min. After rinsing the slides in distilled water and phosphate-buffered saline (PBS), a blocking solution was added for 5 min at room temperature to obviate unspecific hydrophobic bindings between immunoglobulins and fatty tissue or cell membranes. The samples were incubated for 16 h at a temperature of 4 °C with the primary antibodies Gal-8 and Gal-9 (Table 9). By applying a post-block-reagent and the HRP-polymer, the staining was intensified. The substrate-staining with 3,3′-diaminobenzidine chromogen (DAB) was performed and followed by counterstaining with a Mayer acidic hematoxylin. The specimens were dehydrogenated in a rising alcohol series (70%, 96%, and 100%) and Roticlear. Finally, the slides were covered with Roti-Mount (Carl Roth GmBH + Co. KG, Karlsruhe, Germany). Detailed information about the suitable detection system and precise steps are specified in Table 10.

The extent of the expression was evaluated by the immunoreactive score (IRS) by two independent researchers. This semiquantitative score consists of two scales that measure the intensity of the staining (0 = not stained, 1 = low intensity, 2 = medium intensity, 3 = high intensity) and the percentage of the stained tumor cells (0 = 0%, 1 = 1–10%, 2 = 11–50%, 3 = 51–80%, 4 > 80%). Finally, both scales are multiplied, and the IRS has a range from 0 = no expression to 12 = very high expression. The IRS was applied separately in the cytoplasm and nucleus of the tumor cells. Images of the specimens were taken with a color camera (JVC, Victor Company of Japan, Yokohoma, Japan).

### 4.4. Statistical Analysis

IBM SPSS Statistics version 26 (IBM Corp., Armonk, NY, USA,) was used to perform statistical analysis. Bivariate correlations were calculated by Spearman’s-rank-correlation coefficient, and non-parametric tests (NPAR: Mann-Whitney-U test, Kruskal-Wallis test) were performed to compare independent groups. Kaplan-Meier-curves and log-rank-test (Mantel-Cox) were used for survival analysis. In order to identify the independence of prognostic markers, Cox regression analysis was performed. Survival times are shown in months. *p*-values had to be < 0.05 to be considered statistically significant.

## 5. Conclusions

We examined the expression of Galectin-8 and -9 in 225 cases of endometrial cancer. We showed that cytosolic expression of both galectins is associated with grading and survival data. Cytosolic Galectin-8 expression is a positive prognostic factor for OS and PFS, while cytosolic presence of Galectin-9 in endometrial cancer is correlated to a better prognosis regarding overall survival. Further investigation of Gal-8 and Gal-9, including the examination of the different isotypes and the underlying molecular mechanisms in the context of the immune microenvironment could lead to new strategies for targeted therapy.

## Figures and Tables

**Table 1:** Distribution of histopathological parameters.

Item	No./Total No.	%
Age		
<65 years	109/225	48.4
>65 years	116/225	51.6
Tumor Size, pT		
pT1	175/225	77.8
pT2	16/225	7.1
pT3	30/225	13.3
pT4	3/225	1.3
Not available	1/225	0.4
Lymph-Node Status, pN		
N−	142/225	63.1
N+	21/225	9.1
Not available	62/225	27.6
Distant Metastasis Status, pM		
M−	109/225	48.4
M+	5/225	2.2
Not available	111/225	49.3
FIGO		
I	167/225	74.2
II	15/225	6.7
III	36/225	16.0
IV	6/225	2.7
Not available	1/225	0.4
Tumor Grade		
I	127/225	56.4
II	77/225	34.2
III	20/225	8.9
Not available	1/225	0.4
Progression (over 235 months)		
None	180/225	80.0
At least one	45/225	20.0
Survival (over 235 months)		
Right censured	93/244	41.3
Died	132/225	58.7

**Table 2:** Correlation of Gal-8 expression in endometrial cancer to histopathological variables. Significant results are shown in bold; SD = Standard deviation. NPAR = Non-parametric test; n.a. = Not available.

	Gal-8 Cytosolic			Gal-8 Nuclear		
	Median IRS (± SD)	%	*p* (NPAR)	Median IRS (± SD)	%	*p* (NPAR)
pT			0.217			0.509
T1	4 (± 3.32)	15.4		2 (± 2.77)	17.8	
T2	4 (± 2.75)	25.0		2 (± 1.77)	22.1	
T3	4 (± 3.03)	13.3		0 (± 2.85)	46.7	
T4	0 (± 3.46)	66.7		0 (± 3.46)	66.7	
pN			0.266			0.251
N−	4 (± 3.37)	16.2		2 (± 2.81)	13.4	
N+	4 (± 2.55)	23.8		1 (± 3.12)	14.3	
FIGO			0.146			0.067
FIGO I	4 (± 3.38)	13.8		2 (± 2.81)	13.2	
FIGO II	4 (± 2.63)	26.7		2 (± 1.44)	28.6	
FIGO III	4 (± 2.80)	11.1		1 (± 2.72)	13.9	
FIGO IV	2 (± 2.67)	n.a.		0 (± 2.45)	83.3	
FIGO			0.065			0.104
FIGO I	4 (± 3.38)	13.8		2 (± 2.81)	13.2	
FIGO II–IV	4 (± 2.27)	28.1		1 (± 2.40)	08.8	
Grading			**0.029** (Rho: −0.132; *p* = 0.065)			0.077
G1	4 (± 3.18)	17.3		2 (± 2.55)	11.0	
G2/G3	4 (± 3.29)	17.5		1 (± 2.82)	05.2	

**Table 3:** Correlation of Gal-9 expression in endometrial cancer to histopathological variables. Significant results are shown in bold; SD = Standard deviation. NPAR = Non-parametric test; n.a. = Not available.

	Gal-9 Cytosolic			Gal-9 Nuclear		
	Median IRS (± SD)	%	*p* (NPAR)	Median IRS (± SD)	%	*p* (NPAR)
pT			0.516			0.509
T1	4 (± 2.92)	20.0		0 (± 0.66)	79.4	
T2	6 (± 3.09)	18.8		0 (± 0.72)	75.0	
T3	4 (± 3.37)	16.7		0 (± 1.22)	83.3	
T4	2 (± 2.31)	66.7		0 (± 0.00)	100	
pN			0.227			0.057
N−	4 (± 2.93)	20.4		0 (± 0.72)	76.1	
N+	4 (± 3.28)	14.3		0 (± 0.00)	81.0	
FIGO			0.367			0.602
FIGO I	4 (± 2.90)	13.8		0 (± 0.67)	79.0	
FIGO II	6 (± 3.09)	20.0		0 (± 0.72)	80.0	
FIGO III	4 (± 3.50)	19.4		0 (± 1.15)	80.6	
FIGO IV	3.5 (± 2.59)	n.a.		0 (± 0.00)	100	
FIGO			0.389			0.672
FIGO I	4 (± 3.38)	13.8		0 (± 0.67)	79.0	
FIGO II–IV	4 (± 2.27)	28.1		0 (± 0.97)	08.8	
Grading			**<0.001** (Rho: −0.237; *p* = 0.001)			0.599
G1	6 (± 2.85)	19.7		0 (± 0.97)	82.5	
G2/G3	4 (± 2.99)	26.0		0 (±0.65)	76.6	

**Table 4:** Correlation of Gal-8 and Gal-9. CC = Correlation coefficient. Significant results are shown in bold.

		IRS Gal-8 Cytosolic	IRS Gal-8 Nuclear	IRS Gal-9 Cytosolic	IRS Gal-9 Nuclear
IRS Gal-8 cytosolic	CC	1	**0.517**	**0.218**	0.078
	*p*	-	**<0.001**	**0.004**	0.297
IRS Gal-8 nuclear	CC	**0.517**	1	0.076	0.041
	*p*	**<0.001**	-	0.319	0.581
IRS Gal-9 cytosolic	CC	**0.218**	0.076	1	**0.324**
	*p*	**0.004**	0.319	-	**<0.001**
IRS Gal-9 nuclear	CC	0.078	0.041	**0.324**	1
	*p*	0.297	0.581	**<0.001**	-

**Table 5:** COX-Regression Gal-8 expression and OS. Significant results are shown in bold.

Variable	Significance	Hazard Ratio of Exp(B)	Lower 95% CI of Exp(B)	Upper 95% CI of Exp(B)
Age	**<0.001**	1.074	1.037	1.113
pT	0.187	1.631	0.789	3.373
pN	0.808	1.123	0.441	2.865
Grading	**0.042**	1.503	1.041	2.229
FIGO	0.992	0.996	0.447	2.22
Galectin-8 cytoplasm	**0.017**	0.224	0.066	0.763
Galectin-8 nucleus	0.413	0.799	0.467	1.368

**Table 6:** COX-Regression Gal-8 expression and PFS.

Variable	Significance	Hazard Ratio of Exp(B)	Lower 95% CI of Exp(B)	Upper 95% CI of Exp(B)
Age	0.235	1.035	0.978	1.069
pT	0.341	1.487	0.657	3.364
pN	0.874	0.906	0.266	3.089
Grading	0.464	1.279	0.662	2.472
FIGO	0.153	2.007	0.772	5.219
Galectin-8 cytoplasm	0.977	0	0	-
Galectin-8 nucleus	0.866	1.079	0.447	2.606

**Table 7:** COX-Regression Gal-9 expression and OS. Significant results are shown in bold.

Variable	Significance	Hazard Ratio of Exp(B)	Lower 95% CI of Exp(B)	Upper 95% CI of Exp(B)
Age	**<0.001**	1.08	1.041	1.12
pT	0.254	1.622	0.707	3.723
pN	0.131	2.156	0.795	5.844
Grading	0.076	1.431	0.963	2.126
FIGO	0.573	0.774	0.318	1.884
Galectin-9 cytoplasm	0.074	0.55	0.286	1.059
Galectin-9 nucleus	0.914	0.895	0.119	6.723

**Table 8:** COX-Regression Gal-9 expression and PFS.

Variable	Significance	Hazard Ratio of Exp(B)	Lower 95% CI of Exp(B)	Upper 95% CI of Exp(B)
Age	0.687	1.011	0.959	1.066
pT	0.654	1.236	0.489	3.126
pN	0.964	1.031	0.282	3.769
Grading	0.18	1.564	0.814	3.005
FIGO	0.236	1.855	0.668	5.153
Galectin-9 cytoplasm	0.606	0.762	0.271	2.143
Galectin-9 nucleus	0.986	0	0	-

**Table 9:** Primary antibodies.

Antigen	Company	Antibody	Host	Synonyms	Catalog ID
Galectin-8	Abcam	monoclonal IgG	rabbit	clone EPR4857	ab109519
Galectin-9	Abcam	polyclonal IgG	rabbit	-	ab69630

**Table 10:** Overview of antibodies and chemicals used in the staining process.

Galectin-8	Galectin-9
Blocking solution ^1^: 5 min	Blocking solution ^1^: 5 min
primary antibody ^2^: 1:150 in PBS ^4^	primary antibody ^3^: 1:300 in PBS ^4^
incubation 1 h at room temperature	incubation 16 h at 4 °C
PostBlock ^1^: 20 min	Post Block ^1^: 20 min
HRP Polymer ^1^: 30 min	HRP Polymer ^1^: 30 min
Chromogen: DAB ^5^ (1 min)	Chromogen: DAB ^5^ (1 min)

^1^ ZytoChem Plus HRP Polymer Kit (Mouse/Rabbit) 3 × 100; company: Zytomed Systems (Berlin, Germany); ^2^ Anti galectin-8 (rabbit IgG monoclonal, clone EPR4857), concentration not determined, company: Abcam (Cambridge, UK), order number: ab109519; ^3^ Anti galectin-9 (rabbit IgG polyclonal), concentration: 1 mg/mL, company: Abcam (Cambridge, UK), order number: ab69630; ^4^ Liquid DAB + Substrate Chromogen System 1 mg/mL, DAKO; ^5^ 3,3′-diaminobenzidine chromogen.

## Data Availability

All data generated or analysed during this study are included in this published article. The data presented in this study are available on request from the corresponding author.

## Supplementary Materials

The supporting information can be downloaded at: https://www.mdpi.com/article/10.3390/ijms25136907/s1.

## Abbreviations

EC	Endometrial cancer
FIGO	Fédération Internationale de Gynécologie et d‘Obstétrique
Gal-8	Galectin 8
Gal-9	Galectin 9
IRS	Immunoreactive score
MMR	Mismatch repair
NPAR	Non-parametric
n.a.	Not available
OS	Overall survival
PFS	Progression—free survival
POLE	Polymerase ε
ROC	Receiver operating characteristic
SD	Standard deviation
TCGA	The cancer genome atlas
