# Supplementary material for: Expression of Intracellular Galectin-8 and -9 in Endometrial Cancer
Source: Int J Mol Sci. 2024 Jun 24;25(13):6907. doi: 10.3390/ijms25136907 (PMC11241125; doi:10.3390/ijms25136907)
Supplement: Supplementary file 1 [file ijms-25-06907-s001.zip › ijms-3043792-supplementary.pdf]

Supplement S1

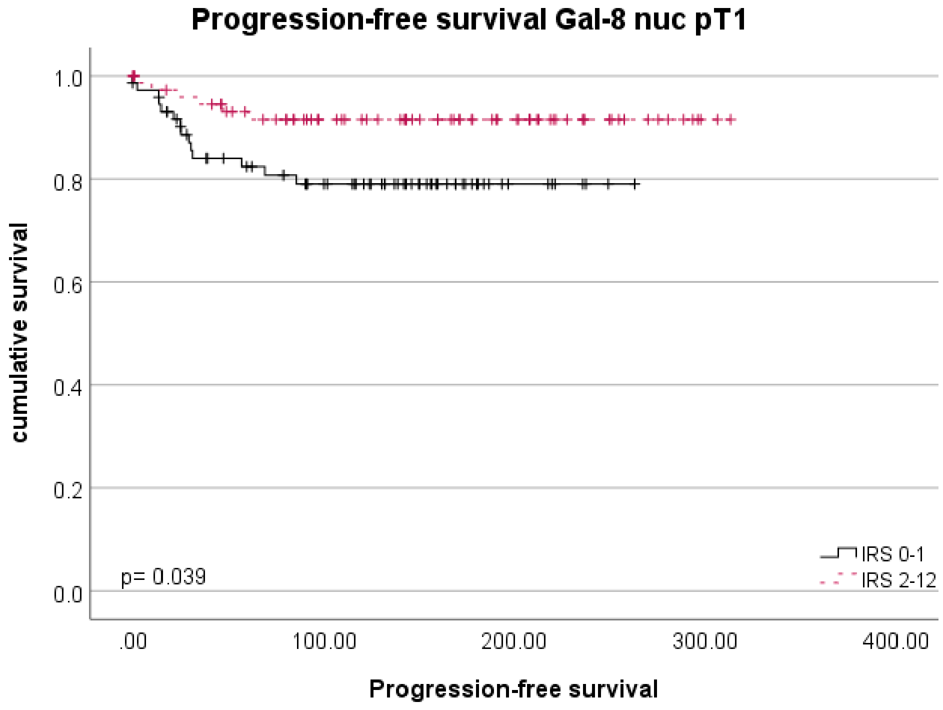

Supplement Figure S1. PFS in subgruoups: significant better PFS with high nuclear Gal-8 expression in the subgroup pT1 (IRS 2-12; p=0.039).

Supplement S2

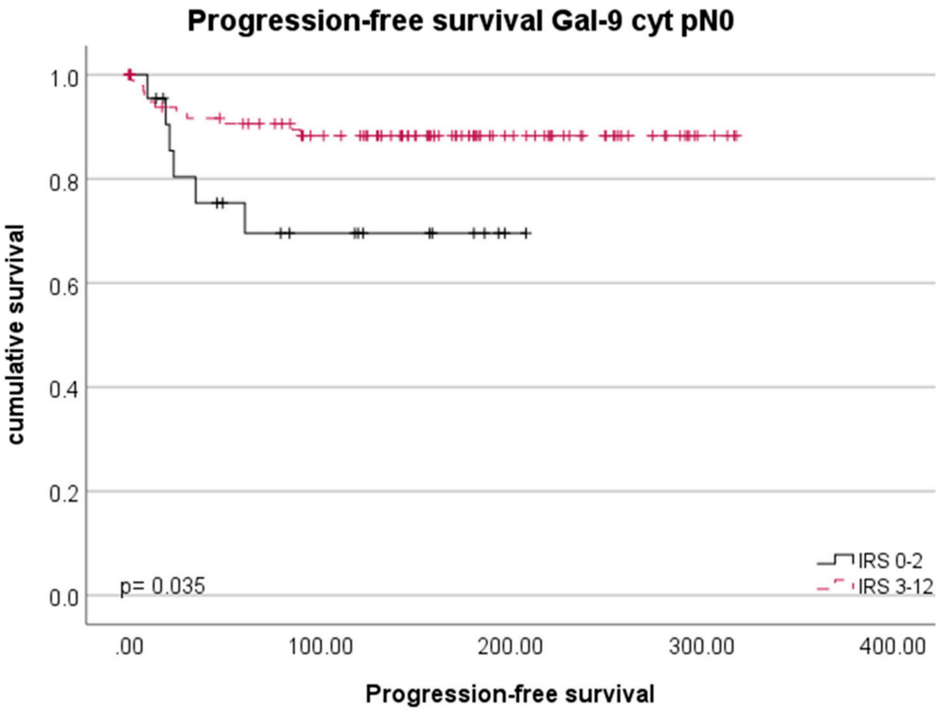

Supplement Figure S2. PFS in subgruoups: significant better PFS with high cytosolic Gal-9 expression in the subgroup pN0 (IRS 3-12; p=0.035).
